# Direct Observation of ATP-Induced Conformational Changes in Single P2X_4_ Receptors

**DOI:** 10.1371/journal.pbio.1000103

**Published:** 2009-05-05

**Authors:** Youichi Shinozaki, Koji Sumitomo, Makoto Tsuda, Schuichi Koizumi, Kazuhide Inoue, Keiichi Torimitsu

**Affiliations:** 1 NTT Basic Research Laboratories, NTT Corporation, Kanagawa, Japan; 2 Department of Molecular and System Pharmacology, Graduate School of Pharmaceutical Sciences, Kyushu University, Fukuoka, Japan; 3 Department of Pharmacology, Interdisciplinary Graduate School of Medicine and Engineering, University of Yamanashi, Chuo, Yamanashi, Japan; Cambridge University, United Kingdom

## Abstract

The ATP-gated P2X_4_ receptor is a cation channel, which is important in various pathophysiological events. The architecture of the P2X_4_ receptor in the activated state and how to change its structure in response to ATP binding are not fully understood. Here, we analyze the architecture and ATP-induced structural changes in P2X_4_ receptors using fast-scanning atomic force microscopy (AFM). AFM images of the membrane-dissociated and membrane-inserted forms of P2X_4_ receptors and a functional analysis revealed that P2X_4_ receptors have an upward orientation on mica but lean to one side. Time-lapse imaging of the ATP-induced structural changes in P2X_4_ receptors revealed two different forms of activated structures under 0 Ca^2+^ conditions, namely a trimer structure and a pore dilation-like tripartite structure. A dye uptake measurement demonstrated that ATP-activated P2X_4_ receptors display pore dilation in the absence of Ca^2+^. With Ca^2+^, the P2X_4_ receptors exhibited only a disengaged trimer and no dye uptake was observed. Thus our data provide a new insight into ATP-induced structural changes in P2X_4_ receptors that correlate with pore dynamics.

## Author Summary

ATP is not only a source of intracellular energy but can act as an intercellular signal by binding membrane receptors. Purinergic receptors, which bind with nucleotides including ATP are known as P2 receptors and are divided into two types: ion channel-type P2X receptors and metabotropic-type P2Y receptors. P2X receptors are thought to undergo conformational changes in response to ATP binding, leading to the opening of transmembrane channels, through which cations enter the cells. A growing body of evidence shows that P2X receptors control various physiological and pathophysiological cellular responses. However, the receptor structure and the conformational changes it experiences upon stimulation remained to be clarified. Here, we employed an atomic force microscope (AFM) to observe P2X receptor behavior at the single channel level. We chose to analyze the P2X4 receptor, because it is known to increase the transmembrane pore size (i.e., pore dilation) in the absence of extracellular calcium. Activated P2X4 receptor exhibited a trimeric topology with a pore-like structure in the center. When calcium was present the receptor exhibited a trimer without a pore structure at its center. These structural changes corresponded well with the changes of ion permeability of P2X4 receptor.

## Abstract 3

Fast-scanning atomic force microscopy reveals the topology, ATP-induced conformational changes, and Ca^2+^ regulation of the pore-opening in P2X_4_ receptors.

## Introduction

P2X receptors (P2XRs) are cell-surface ATP-gated cation channels, and seven subtypes (P2X_1–7_) are known [1]. One functional P2XR channel is composed of three subunits. Each P2XR subunit is predicted to have a large extracellular domain (ECD), two transmembrane-spanning domains (TMD), and N and C termini intracellular domains (ICD) [1]. It has been suggested that the second half of the ECD (residues 170–330) has sequence and secondary structure similarities to the catalytic site of class II aminoacyl-tRNA synthetase [2]. A six-stranded antiparallel β-pleated sheet structure is believed to exist in the ECD of P2XRs. 3-D homology modeling in P2X_4_Rs suggests that this region coordinates ATP binding and the allosteric coupling of the conformational changes in the ATP binding domain with corresponding changes at the transmembrane channel gate through a linker region (the α-helix between the β6 strand and TM2 region) [3]. In addition to the allosteric coupling of the ATP-binding sites at ECDs and the channel gate at TMD, P2XRs have different permeability states that were originally discovered by Cockcroft and Gomperts [4]. With P2X_4_Rs, extracellular Ca^2+^ levels greatly affect the permeability dynamics [5]. In the presence of Ca^2+^, P2X_4_R only opens a small cation-permeable channel pore but in the absence of extracellular Ca^2+^ it forms a larger pore that allows larger molecules including *N*-methyl-D-glucamine (NMDG)^+^, propidium iodide, and ethidium bromide (EtBr) to pass. Although there is a functional relationship between ECD and TMD, the ATP-induced structural changes in ECD are poorly understood. Recent extensive studies by Khakh's group have clearly demonstrated the allosteric coupling of ICDs and the ion channel permeability of P2XRs [6,7]. These results strongly support the hypothesis of the allosteric coupling of channel pores in TMD and other domains including ECDs.

In recent structural studies of P2XRs two approaches have been used: electron microscopy (EM) and atomic force microscopy (AFM). In EM, single particle averaging analysis and the Ni-NTA gold labeling of human P2X_4_Rs have clearly demonstrated the distance between the C-terminal tails, the molecular volume, and the 3-D structure [8]. In AFM research, an antibody tagging study has revealed the trimer structure of P2XRs [9,10]. AFM has the important advantage of allowing proteins to be observed under liquid conditions, and this makes it possible to activate P2XRs by ATP during AFM studies. In an AFM study combined with ATP treatment, P2XRs exhibited a pore-like structure [11]. In addition to drug treatment, AFM can be used for imaging both lipid bilayers [12] and proteins inserted in lipid membranes [13]. Extensive AFM studies by Engel and Müller's groups have obtained high-resolution topographs of many proteins including aquaporin [14], connexin [15], F-ATP synthase [16], and tubulin [17]. A recent study by Cisneros clearly demonstrated the topography of orientation regulated and covalently assembled homotrimer OpmF proteins [18]. In their report, the authors employed the single particle correlation averaging method to obtain 3-fold symmetrized images of OmpF trimer that are identical to the topographs of 2-D crystals of OmpF. Because many P2XR channels are also homotrimers, this approach can be used for the high-resolution imaging of P2XRs. Although the use of AFM provides significant advantages the imaging speed is usually very slow (several tens of seconds). Many ion channel reactions occur in less than a second, so fast scanning is essential for observing the P2XR reaction with AFM. To address this issue, we employed a recently developed fast-scanning AFM [19] that allows us to observe biological molecules including nucleic acids [20], lipids [12], and proteins [21,22] at high temporal resolution. Fast-scanning AFM in combination with single particle averaging is considered a powerful tool for analyzing single P2X_4_R channels with high spatial and temporal resolution.

## Results

### Expression, Purification, and AFM Observation of P2X_4_Rs on Poly-D-Lysine-Coated Mica

**Figure 1:**
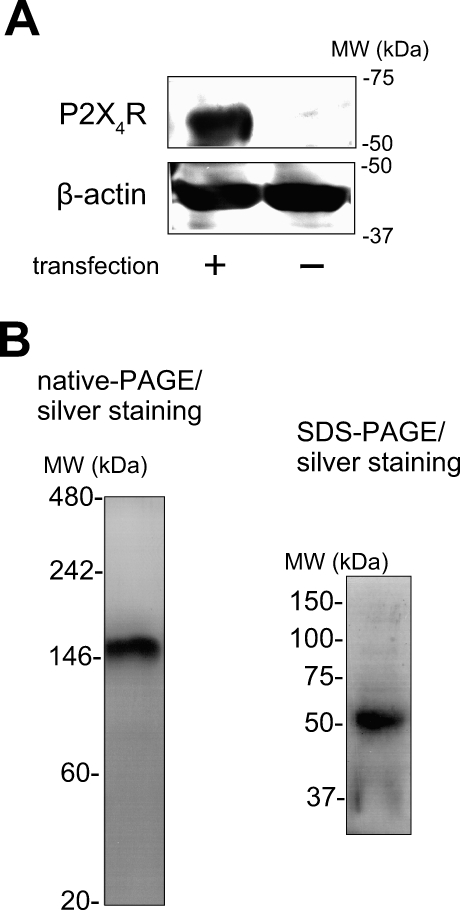
Expression and Purification of P2X_4_R (A) Overexpression of P2X_4_R in human 1321N1 astrocytoma cells. In Western blotting, P2X_4_R protein was only detected in cells transfected with the P2X_4_R gene. (B) Purification of P2X_4_R protein. The molecular weight of the purified P2X_4_R protein band was detected at about 150 kDa in native-PAGE (left) and 50 kDa in SDS-PAGE (right). After electrophoresis, the gels were stained with silver staining.

**Figure 2:**
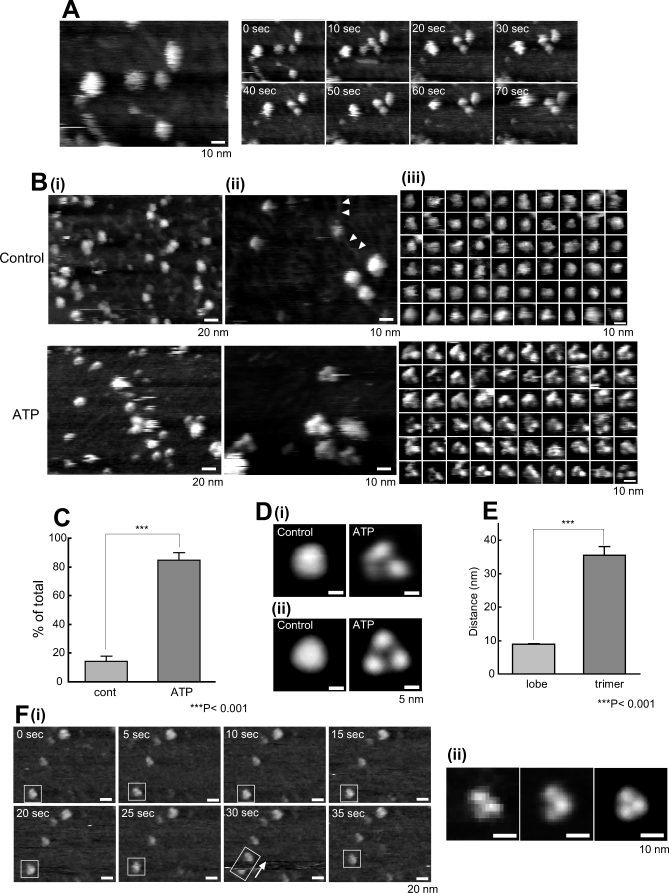
AFM Observations of P2X_4_Rs on PDL-Coated Mica (A) P2X_4_Rs attach stably to PDL-coated mica. P2X_4_Rs on PDL-coated mica exhibited stable attachment and the majority did not shift position during AFM observation. Scale bar, 10 nm. (B) AFM images of P2X_4_Rs at (i) low resolution, (ii) high resolution, and (iii) single particle level. (i) At low resolution, the P2X_4_Rs were relatively homogenous. Slight differences were observed after ATP treatment (1 mM, 30 min), but they are not very clear at this resolution. Scale bar, 20 nm. (ii, iii) At high resolution and at a single particle level, there were significant structural differences between the control P2X_4_Rs and the P2X_4_Rs after ATP addition. Each single particle was selected based on our criteria (Materials and Methods, Figure S1C). In the control, the P2X_4_Rs were nearly circular, ellipsoid, or triangular with obtuse angles. After ATP addition, the P2X_4_Rs had a tripartite morphology. PDL-polymers were also observed (arrows). Scale bar, 10 nm. (C) Percentage of trimeric P2X_4_R was significantly increased after ATP (1 mM, 30 min). ***, *p* < 0.001. (D) Averaged images of P2X_4_Rs. (i) Nonsymmetrized averaging of P2X_4_Rs in the control (left) and after ATP addition (right). (ii) Symmetrized averaging of P2X_4_Rs in the control (left) and after ATP addition (right). 3-fold symmetrized images were obtained after symmetrized averaging. Scale bar, 5 nm. (E) Three lobes are individual subunits in one P2X_4_R trimer. The distance between lobes was significantly less than that between trimers. ***, *p* < 0.001. (F) P2X_4_R trimer shifts position as one unit. (i) When a P2X_4_R trimer moves during AFM, the three lobes were not dissociated but moved as a trimer. Scale bar, 20 nm. (ii) Enlarged images of single P2X_4_R trimer in a rectangle at 5 s, nonsymmetrized and symmetrized averaging images of ten particles in the same scan area. Scale bar, 10 nm. AFM observation was performed in AFM imaging buffer A.

The expression of rat P2X_4_R protein in human 1321N1 astrocytoma cells was estimated by western blotting. P2X_4_R was detected only in P2X_4_R gene-overexpressed cells (Figure 1A). In silver-stained native PAGE, only one band corresponding to a trimer (about 150 kDa) (Figure 1B) was observed. The same protein analyzed by SDS-PAGE and silver-staining exhibited a band corresponding to a monomer (about 50 kDa). For the AFM analysis of P2X_4_Rs, we used freshly cleaved mica as a substrate because it has an atomically flat surface and is usually used for protein observation with AFM. All the AFM images were presented as gray-scale height images. In many cases, the P2X_4_R particles were only loosely attached to the uncoated mica and so they moved during the AFM observation. To obtain a stronger attachment for electrostatic interactions, we coated the mica with positively charged poly-D-lysine (PDL) (1 mg/ml, 30 min at room temperature [RT]) and set the pH of the imaging buffer (AFM imaging buffer A) at 8.0 because the isoelectric point of P2X_4_R is pH 7.41. All the P2X_4_Rs on the PDL-coated mica were observed in AFM imaging buffer A. Under this condition, the P2X_4_Rs were attached stably to the substrate (Figure 2A). The P2X_4_R control particles were relatively homogenous and nearly all circular, ellipsoid, or triangular with obtuse angles (Figure 2B, upper panels). PDL-polymers were also observed (Figure 2B[ii], arrows). In this study, we defined the dimensions of the P2X_4_Rs as their diameter and height on the basis of our criteria (see also Materials and Methods and Figure S1). The nonstimulated P2X_4_Rs had a diameter of 12.6 ± 0.2 nm (mean ± standard error of the mean [SEM]) (*n* = 200) and a height of 2.3 ± 0.1 nm. To observe activated P2X_4_Rs, we added ATP before the AFM observation. ATP did not induce any significant changes at 100 μM (unpublished data), but the P2X_4_Rs changed greatly at 1 mM (Figure 2B, lower panels). Under this condition, at least several minutes of ATP treatment was required before the P2X_4_Rs underwent structural changes. After the structural changes caused by 1 mM ATP, most of the P2X_4_Rs appeared to be trimers (84.9 ± 5.0%, *n* = 393) (Figure 2C). The ATP-treated P2X_4_Rs had a diameter of 14.2 ± 0.2 nm (*n* = 205) and a height of 3.0 ± 0.1 nm. The diameter of one lobe in a P2X_4_R trimer was 5.9 ± 0.2 nm (*n* = 40). To obtain clear topographs of P2X_4_Rs, we averaged single P2X_4_R images by using the same approach employed by Cisneros et al. [18] and on the basis of our criteria (Figure S1). The nonsymmetrized averaging of ATP-treated P2X_4_Rs revealed a tripartite morphology (Figure 2D[i], right) that was enhanced by 3-fold rotational symmetrization (Figure 2D[ii], right). Nonstimulated P2X_4_Rs were circular or triangular with obtuse angles after averaging (Figure 2D, left panels). For averaging, we used the particles shown in Figure 2B(iii) (*n* = 60). Then, we checked whether these trimers were one unit of P2X_4_R trimers or simply three adjacent particles. If each lobe was an individual P2X_4_R trimer that was incidentally assembled into a trimer, the distance between lobes would not be significantly different from the distance between trimers. The distance between the lobes in a P2X_4_R trimer and the distance between two adjacent trimers were 8.7 ± 0.1 nm (*n* = 100, between lobes) and 35.5 ± 2.7 nm (*n* = 115, between trimers), respectively (Figure 2E). Sometimes, P2X_4_R particles on PDL-coated mica shifted position within the same scan area. In this situation, single lobes in a P2X_4_R trimer (15 min after 1 mM ATP treatment) did not move individually but moved along with the other two lobes (Figure 2F[i]). Enlarged images of single P2X_4_R trimer in a rectangle at 5 s are shown on the left in Figure 2F(ii). The nonsymmetrized and symmetrized averaging of ten particles in the same scan area at 0 s is shown in the center and on the right, respectively, in Figure 2F(ii).

### Time-Lapse Imaging of ATP-Induced Structural Changes in Single P2X_4_R

**Figure 3:**
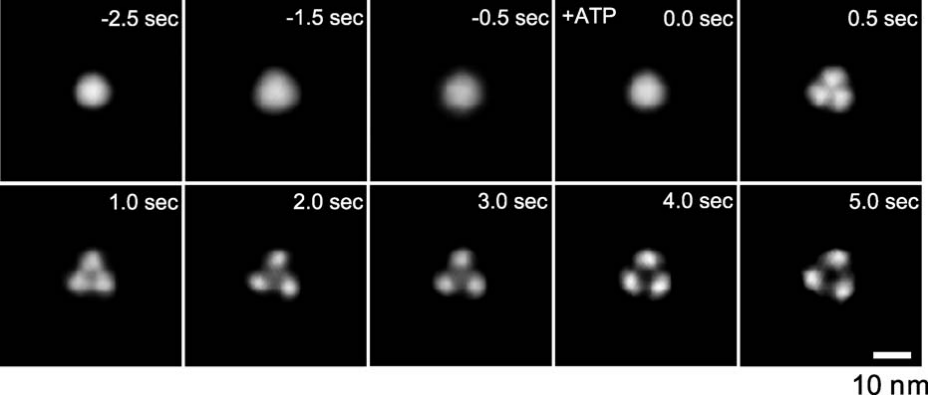
Fast-Scanning AFM Observations of the ATP-Induced Structural Changes in the P2X_4_Rs Time-lapse imaging of ATP-induced structural changes of P2X_4_R. Before activation, P2X_4_R was in circular shape and exhibited some fluctuation (−2.5 s to ≈0.0 s). Caged ATP (200 μM) was uncaged at 0 s. After uncaging, the P2X_4_R structure changed to a trimer structure within 0.5 s. Then, P2X_4_R exhibited a further structural change and adopted a pore dilation-like conformation. Ten P2X_4_R particles were averaged for each frame. Scale bar, 10 nm. AFM observation was performed in AFM imaging buffer B.

To observe the ATP-induced continuous structural changes in P2X_4_Rs, we performed imaging using fast-scanning AFM with a scan rate of two frames per second. P2X_4_Rs were observed in AFM imaging buffer B. Under our conditions, faster scan rates than this degraded the signals and increased noise so that we were unable to obtain sufficient resolution. It is known that a mica surface is negatively charged [23], and so we used uncoated mica rinsed with a high concentration of KCl (1 M, 30 min at RT) to reduce electrostatic interactions between the mica surface and the ATP or P2X_4_Rs. Under this condition, many P2X_4_Rs shifted position during AFM imaging. To obtain a clear topology of P2X_4_R, ten P2X_4_R particles were averaged at the same time point. The resulting 3-fold symmetrized images of P2X_4_Rs clearly exhibited the structures at each time point. Before the uncaging (−2.5 to ∼0.0 s) of caged ATP (200 μM), P2X_4_R exhibited a circular structure (Figure 3, see also Video S1). At 0.5 s after uncaging, the P2X_4_R structure changed greatly and a clear trimeric structure was observed. After this change, the distances between individual lobes gradually increased (≈5 s). The conformational change in the nonsymmetrized P2X_4_R is also shown in Figure S2. The same reaction was reproduced in three independent experiments. Another result of the ATP-induced structural changes in P2X_4_R is shown in Figure S3. Some P2X_4_Rs were stable at one location during AFM imaging. Several examples of ATP-induced structural changes in a single P2X_4_R are shown in Figure S4. At a single particle level, although the P2X_4_R topologies were relatively blurry, individual subunits became visible after uncaging and appeared to move away from each other. When the ATP was washed off, the pore dilation-like structure returned to a circular structure (unpublished data).

### AFM Observation and Functional Analysis of Membrane-Inserted P2X_4_Rs

**Figure 4:**
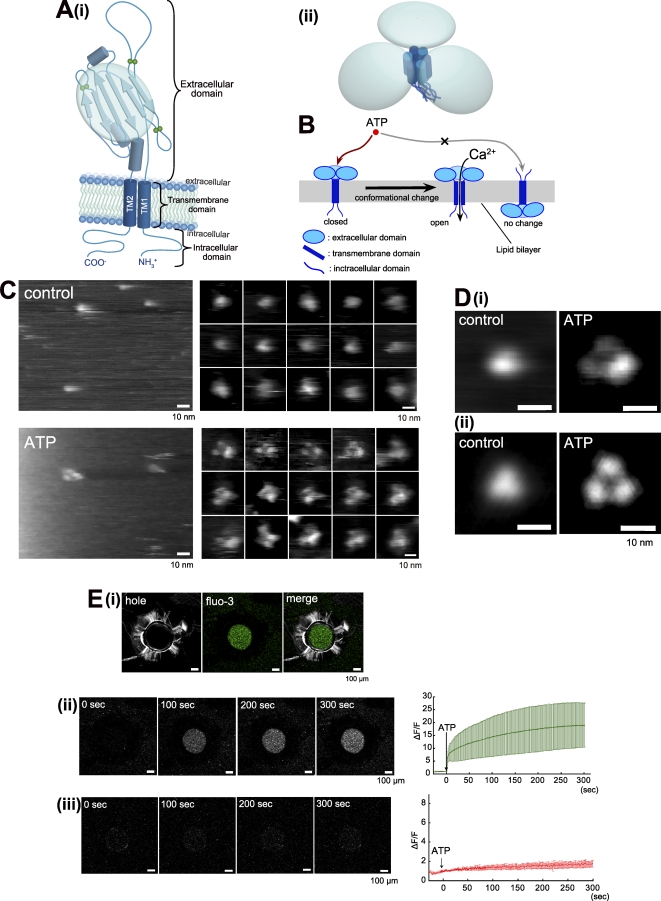
AFM Observation and Functional Analysis of Membrane-Inserted P2X_4_Rs (A) Diagrams illustrating domain structures in (i) one P2XR subunit and (ii) P2XR trimer. The ECD of a P2XR subunit is assumed to have a six-stranded antiparallel β-pleated sheet structure. Three characteristic ECDs are assumed to exist in a P2XR trimer. (B) Hypothetical view of P2X_4_Rs reconstituted in a lipid bilayer. If P2X_4_R were inserted upwardly, ATP-induced structural changes and the Ca^2+^ flow of P2X_4_R would be observed. (C) AFM images of membrane-inserted P2X_4_Rs. In the control, the P2X_4_Rs were homogenous and largely circular, ellipsoid, or triangular with obtuse angles (upper panels). After ATP addition (200 μM, 1 min), the P2X_4_Rs exhibited tripartite morphologies (lower panels). (D) Averaged images of membrane-inserted P2X_4_Rs. (i) Nonsymmetrized averaging of P2X_4_Rs in the control (left) and after ATP addition (right). (ii) Symmetrized averaging of P2X_4_Rs in the control (left) and after ATP addition (right). Scale bar, 10 nm. AFM observation was performed in AFM imaging buffer B. (E) Ca^2+^ and dye-uptake imaging of P2X_4_Rs. (i) Green fluorescence derived from fluo3/Ca^2+^ after ATP (100 μM, at 0 s) addition was detected only in the hole made in the plastic plate. (ii, iii) Simultaneous recording of ATP-induce Ca^2+^ permeability and EtBr uptake of P2X_4_R. Under 2 mM Ca^2+^ conditions, the green fluorescence intensity immediately increased after ATP addition (see also Video S2). There was no significant increase in red fluorescence (DNA/EtBr) intensity. Each trace is the mean ± SEM of five independent experiments. Scale bar, 100 μm.

To estimate the orientation of observed structures, P2X_4_Rs were reconstituted in a lipid bilayer. Figure 4A(i) is a diagram showing the predicted structure of a P2X_4_R subunit. A six-stranded anti-parallel β-plated sheet structure is reported to exist in the second half of the ECD in P2X_4_R subunits [2,3]. The entire structure of trimeric P2X_4_R is predicted on the basis of this homology modeling data, as shown in Figure 4A(ii). In AFM, this β-plated sheet structure should be observed as one large domain. Figure 4B shows our working hypothesis, which is that when P2X_4_Rs are reconstituted in a lipid bilayer and if they are inserted in an upward orientation, they should respond to ATP thus resulting in structural changes and increased Ca^2+^ permeability. When P2X_4_Rs were inserted in the lipid bilayer that formed on mica, the AFM images of P2X_4_Rs in membranes were similar to the P2X_4_Rs that were dissociated from the membrane. The P2X_4_Rs had circular structures in the control and trimeric structures after binding with ATP (200 μM, 1 min) (Figure 4C and 4D). P2X_4_Rs reconstituted in a lipid bilayer did not require as high a concentration of ATP as those on PDL-coated mica. Under this condition, the structures of most of the P2X_4_Rs (83.3 ± 5.4%, *n* = 70) changed into a tripartite form. The P2X_4_Rs in the membranes were 11.4 ± 0.3 nm in diameter and 5.8 ± 0.1 nm high (including the height of the membrane) in the control (*n* = 50) and 13.3 ± 0.3 nm in diameter and 6.1 ± 0.1 nm high after ATP addition (*n* = 100). The calculated height of the membrane was 4 nm. The AFM imaging of membrane-inserted P2X_4_Rs was performed in imaging buffer B.

For calcium imaging, the P2X_4_Rs were reconstituted in a lipid bilayer that was suspended over a 500 μm hole. The green fluorescence intensity of fluo-3 (50 μM, in hole) was significantly increased after ATP (100 μM) addition (Figure 4E). This green florescence was only detected in the hole (Fig. 4E[i]). The intensity of the green fluorescence increased rapidly for a few seconds after ATP addition and then increased gradually (Fig. 4E[ii], see also Video S2). The averaged trace was obtained from five individual experiments. An EtBr-dye uptake measurement was performed at the same time as the Ca^2+^ imaging. Here, no increase was observed in red fluorescence after ATP addition (Fig. 4E[iii]). The Ca^2+^ imaging was performed in Ca^2+^ imaging buffer.

### Pore Dilation-Like Structural Changes and Dye Uptake of P2X_4_Rs

**Figure 5:**
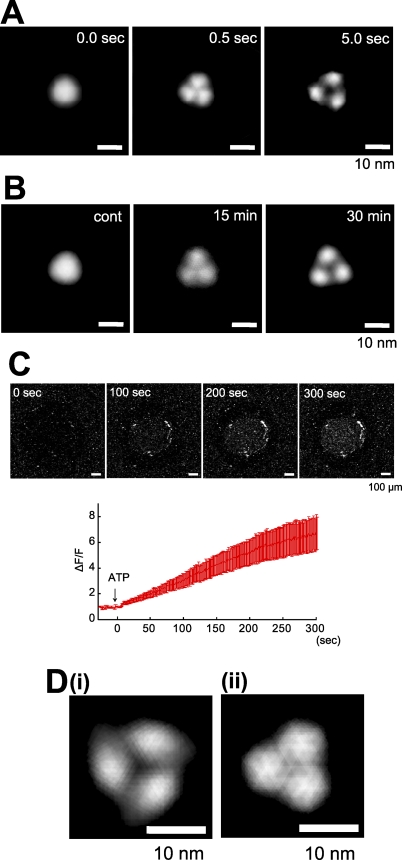
Pore Dilation-Like Structural Changes Are Related to the Dye-Uptake of P2X_4_R (A) Pore dilation-like structural change of P2X_4_R on mica without coating. P2X_4_R was a trimer immediately after activation (0.5 s, 200 μM ATP, middle) and had a pore dilation-like structure 5 s after ATP binding (right). (B) Pore dilation-like structural change of P2X_4_R on PDL-coated mica. After 30 min of ATP (1 mM) treatment, P2X_4_R exhibited a pore dilation-like structure (right) but it had a tripartite topology without a pore dilation-like structure after 15 min of ATP treatment (middle). The AFM observation was performed in AFM imaging buffer B. Scale bar, 10 nm. (C) With 0 mM Ca^2+^, the red fluorescence (DNA/EtBr) intensity gradually increased after ATP addition (see also Video S3). Each trace is the mean ± SEM of five independent experiments. Scale bar, 100 μm. (D) AFM images of activated P2X_4_R (5 s uncaging) under (i) 0 mM and (ii) 2 mM Ca^2+^ conditions. With Ca^2+^, P2X_4_R did not exhibit a pore dilation-like structure. Scale bar, 10 nm.

In the time-lapse imaging of ATP-induced structural changes in P2X_4_R, we observed a characteristic pore dilation-like structure (Figure 3, ≈5.0 s). This pore dilation-like structure was also observed in membrane-reconstituted P2X_4_Rs (Figure 4D). Before the appearance of this structure, the P2X_4_Rs on the uncoated mica exhibited nondilated trimer structures (Figure 5A, center). We observed two P2X_4_R structures similar to these two different forms on PDL-coated mica (Figure 5B). 15 min after ATP (1 mM) addition, the P2X_4_Rs exhibited a nondilated trimer structure (Figure 5B, center) but they exhibited a pore dilation-like structure 30 min after ATP addition (Figure 5B, right). Then we estimated the dye uptake function of P2X_4_Rs using the same Ca^2+^ imaging system. EtBr-uptake imaging buffer containing no Ca^2+^ was used for this study. Here, ATP (100 μM) addition increased the red fluorescence intensity in the hole (Figure 5C, upper panels, see also Video S3). Under our conditions, the red fluorescence intensity started increasing within seconds of the ATP addition and then increased gradually (≈300 s) (Figure 5C, lower panel). When we measured dye uptake with 2 mM Ca^2+^ in an external solution, we observed no increase in red fluorescence intensity (Figure 4E[iii]). To confirm whether the effect of Ca^2+^ on dye uptake is related to the pore dilation-like structural changes, we compared the structures of P2X_4_Rs in the presence and absence of Ca^2+^. In this study, we used the same mica as we used for the time-lapse imaging, and we used AFM imaging buffer B or C for each condition. With 0 Ca^2+^, an averaged P2X_4_R image was obtained from 18 particles (Figure S5A[i]). The particles were selected from frames at least 5 s after uncaging. In this case, a pore dilation-like image was again obtained (Figure 5D[i]). In the presence of 2 mM Ca^2+^, no pore dilation-like averaged image was obtained but a nondilated trimer was observed (Figure 5D[ii]). An averaged P2X_4_R image was obtained from 18 particles (Figure S5A[ii]) at least 5 s after uncaging. The averaged images are obtained after 3-fold symmetrized averaging. Under both conditions, the majority of the P2X_4_Rs responded to ATP (0 Ca^2+^: 67.0 ± 2.8 %, *n* = 257; 2 mM Ca^2+^: 62.8 ± 2.7 %, *n* = 324). Nonsymmetrized averaging images of P2X_4_R under each condition are shown in Figure S5B.

**Figure 6:**
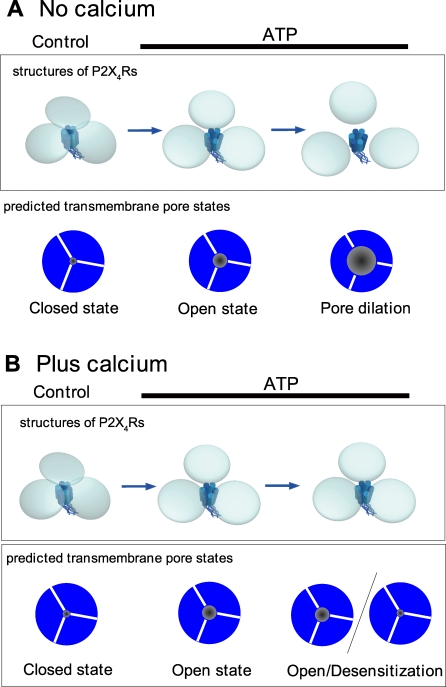
Model of Structural Changes of ECDs in P2X_4_R and Corresponding Pore States Based on AFM and Functional Analysis Data In the control, the ECDs are close to each other and so individual ECDs (or subunits) cannot be observed with AFM. Immediately after ATP binding, three ECDs are disengaged and the TMD pore is Ca^2+^ permeable. (A) In the absence of Ca^2+^, the distances between the ECDs are further increased and the TMD pore becomes EtBr permeable (right). (B) With 2 mM Ca^2+^, P2X_4_R does not exhibit any further structural changes after the disengagement of the ECDs and exhibits no permeability changes.

Models of the ATP-induced structural changes of P2X_4_R based on our results are shown in Figure 6. In the control, three ECDs of P2X_4_Rs were close to each other and AFM revealed no individual subunits. Under this condition, neither Ca^2+^ nor EtBr can permeate the TMD pore. In the absence of Ca^2+^, the ECDs are disengaged and a tripartite topology was observed immediately after ATP binding (Figure 6A, center). Prolonged ATP treatment induces further disengagement of the three ECDs (Figure 6A, right). These two structures appear to correspond to the Ca^2+^ permeable and EtBr permeable states (Figure 6A, below). Under a 2-mM Ca^2+^ condition, P2X_4_R has a nondilated trimer structure regardless of the ATP exposure time (Figure 6B). In this situation, the TMD pores allow Ca^2+^ to permeate but not EtBr however it is unclear whether or not P2X_4_R is desensitized during ATP exposure.

## Discussion

Our main findings in this study are that (i) it is possible to achieve time-lapse imaging of the dynamic structural changes of P2X_4_Rs evoked by ATP; (ii) the three subunits are close to each other and it is impossible to observe individual subunits in the control but they disengage and move away from each other after ATP binding; and (iii) the two types of structural changes observed in AFM appear to correspond to two functional states, namely the Ca^2+^ permeable state and the dye permeable state.

Recent structural studies with direct imaging methods including EM and AFM or with other methods including fluorescence resonance energy transfer (FRET)-based analysis have provided strong motivation for structural studies of P2XRs. These reports clearly demonstrated trimeric stoichiometry using antibody-tagging [9,10] or Ni-NTA gold labeling on the His-tag of the C termini in P2XRs [8], and the shape, architecture, and size of P2X_4_Rs in a nonstimulated state and the distance between the C termini of P2XRs [8]. We needed to determine the way in which P2XRs change their entire structure in response to ATP binding. To address this issue we analyzed homotrimeric P2X_4_Rs. To this end, we overexpressed P2X_4_R gene in human 1321N1 astrocytoma cells. Because this cell does not express P2XRs [24], purified P2X_4_Rs from the membrane fraction of this cell are considered to form homotrimers. In fact, the purified P2X_4_R presented as a single band corresponding to a trimer (about 150 kDa) in native-PAGE but as a monomer (about 50 kDa) in SDS-PAGE, and purified P2X_4_R was functional as estimated in terms of Ca^2+^ permeability. We then observed P2X_4_Rs on mica but they did not attach to it stably. P2X_4_Rs on PDL-coated mica exhibited stable attachment but a high concentration of ATP was required to induce structural changes. The ATP has negative charges that may induce the strong attraction of ATP to the positively charged PDL. In fact, P2X_4_Rs reconstituted in a lipid bilayer or on mica without PDL coating responded to lower ATP concentrations. In addition to the high ATP concentration, a long period of ATP exposure was required when P2X_4_Rs were adsorbed on PDL-coated mica. This may be due to the strong attachment of P2X_4_Rs to mica. In a recent report, the N-terminal tagging of fluorescent proteins on P2X_2_Rs dramatically increased the ATP EC_50_ value, but this did not occur with small tetracystein (4C) tags labeled with fluorescein arsenical hairpin [7], implying that the limited spatial flexibility in the N-terminal domain of P2XRs may reduce the response to ATP. Koshimizu et al. have reported that the cytoplasmic intersubunit interaction prior to ATP binding in P2X_2_R contributes to the subsequent channel activity and conformational changes [25]. The strong attachment of P2X_4_R to mica may also affect the intersubunit interaction via the ICDs, which perhaps causes the reduced response of P2X_4_R to ATP. Under our conditions, the strong attachment of P2X_4_R may change the structural flexibility and/or the intersubunit interaction that reduces the responsivity to ATP. The reduced attachment of P2X_4_Rs to mica without PDL dramatically increased the velocity of the ATP response, and thus supported our hypothesis.

Despite the low ATP reactivity of P2X_4_Rs on PDL, we observed clear structural differences between the control and the ATP-treated condition. We believe that the three lobes observed after ATP addition were three individual subunits of one P2X_4_R trimer. First, the distance between the lobes was significantly smaller than that between trimers. If each lobe was an individual P2X_4_R trimer that was incidentally assembled into a trimer, the distance between lobes would not be significantly different from the distance between trimers. Second, during the AFM observation, some P2X_4_R trimers occasionally shifted position, and these trimers moved as trimers (i.e., the three lobes did not dissociate). Third, in time-lapse analysis, the circular structure changed into a trimer after ATP treatment both in the averaged particle images and in single particles. This result also suggests that the trimeric stoichiometry exists even in P2X_4_Rs before ATP binding. From this observation, we considered circular particles without individual subunits before ATP binding to be trimeric P2X_4_Rs because those subunits were closer together than the spatial resolution of our AFM system. If this is the case, the diameter of the P2X_4_R in the control should be double that of one lobe. In fact, the diameter of the P2X_4_R in the control (about 12.6 nm) was approximately double that of one lobe (5.9 × 2 nm). These three lobes were also observed when P2X_4_Rs were inserted into a lipid bilayer, suggesting that these lobes are the predicted six-stranded antiparallel β-pleated sheet structures in the ECDs of P2X_4_Rs. EM analysis of P2X_4_Rs revealed propeller-like domains in the ECDs [8] that were similar to the six-stranded antiparallel β-pleated sheet-like structure that we observed in the ECDs. In their report, the authors clearly demonstrated that the EM-based distance between the C termini of the P2X_4_Rs was 6.1 nm and the FRET-based distance between the C termini was 5.6 nm. The three propeller-like domains at the opposite end of the P2X_4_R to the gold-labeled C termini means the distances between these domains would be similar. As described above, when three lobes are assembled close together in the control, the distance between the centers of two lobes is twice the lobe radius (2.95 × 2 nm), which agrees well with the distance between P2X_4_R C termini estimated by FRET and EM [8]. As mentioned above, the AFM images of P2X_4_Rs in a lipid bilayer and on mica were comparable; this result strongly suggests the upward direction of the P2X_4_Rs on mica. However, the height of P2X_4_R on mica was less than the height of a lipid bilayer composed of phospholipids (about 4 nm) [12]. In nonsymmetrized averaging, one of the three lobes in the P2X_4_R trimer on mica was lower than the other two. The height of the P2X_4_Rs on mica was only slightly greater than that from the surface of a lipid bilayer to the top of the inserted P2X_4_Rs, indicating the possibility that the P2X_4_Rs do not stand vertically and TMD and/or ICD are bent during the AFM observation. From these observations, we concluded that P2X_4_Rs lean to one side on mica and TMD or ICD may be bent and concealed behind the ECDs. Similarly, the simple adsorption of P2X_2_Rs [11] on mica also results in these molecules having a top view-like structure in AFM images, thus supporting our conclusions.

In the time-lapse imaging, we observed two different structural changes: (i) from one circular structure to a trimeric structure (0.0 → 0.5 s after uncaging) and (ii) the subsequent moving away of each lobe (0.5 → 5.0 s). The second structural change reminds us of an important function of the P2X family, namely pore dilation. In an early study, Khakh et al. clearly demonstrated that P2X_4_R exhibits NMDG^+^ permeable pore dilation in the absence of extracellular Ca^2+^ [5]. In their work, the P2X_4_Rs exhibited sustained activity for several minutes, indicating that our pore dilation-like structure is not a desensitized P2X_4_R state. Our work represents direct evidence of the functional and structural relationship of pore dilation in P2X_4_R. Under a 0 Ca^2+^ condition, we observed both pore dilation-like structural changes in ECDs and EtBr uptake. This pore dilation-like change was reproducible under various conditions including on mica, on PDL-coated mica and in a lipid bilayer, strongly suggesting that this structural change is a fundamental reaction of P2X_4_R. At 2 mM Ca^2+^, we observed no EtBr uptake but there was a Ca^2+^ flow via P2X_4_R that also corresponded to the previous report [5]. Under this condition, the pore dilation-like structure of P2X_4_R was not observed but P2X_4_R trimers similar to the structure seen immediately after ATP binding were evident. The averaged trace of the green fluorescence intensity exhibited a near-plateau state after an initial increase. This result may indicate that the number of desensitized P2X_4_Rs increase during a long ATP exposure. From these observations, we considered that the structural changes in the ECDs of P2X_4_Rs are related to permeability dynamics. Recent reports on P2X_7_Rs suggested the possibility that their EtBr uptake is mediated by accessory Pannexin-1 (Panx1) channels [26]. In their report, the authors demonstrated that human 1321N1 cells express Panx1, so it is possible that there is functional coupling between overexpressed P2X_4_Rs and Panx1 in this cell. We concluded that EtBr can pass through P2X_4_R independent of Panx1 (at least in our study) for the following reasons. First, we used purified P2X_4_Rs and only a single band was observed in the native-PAGE/silver staining. As Panx1 (about 50 kDa) forms a hexameric channel [27], Panx1 contamination would be detected as another band (about 300 kDa). Second, Panx1 and connexins are known to have structural similarities [28] and connexins are observed as hexameric structures [15] in AFM. We observed no hexameric structures in our AFM study. Third, the issue of Panx1 coupling with P2X_7_R remains to be clearly settled because another group has demonstrated that P2X_7_R exhibits pore dilation independent of Panx1 [29]. P2X_2_R also exhibits the pore dilation independent of Panx1 [7]. These results indicate that Panx1 may not be a fundamental component of the pore dilation state of the P2XR family. Fourth, in contrast to connexin hemichannels, Panx1 is active at physiological extracellular Ca^2+^ concentrations [28]. In our simultaneous Ca^2+^/dye uptake measurement, EtBr uptake was not observed at 2 mM Ca^2+^. However, our data and these reports do not rule out the possibility of functional coupling between P2X_4_Rs and Panx1 in cells.

Thus, our present study provides direct evidence of structural changes in the ECDs of P2X_4_Rs that are involved in permeability dynamics. We have achieved the first direct, time-lapse imaging, to our knowledge, of ATP-induced structural changes of P2X_4_R using a new technique, namely fast-scanning AFM. Our approach provides new insights into the structure of P2XRs, and an extension of this approach to other P2X subtypes will help us to understand the structural and functional relationships of the P2XR family.

## Materials and Methods

### Reagents.

Reagents were obtained from the following sources. DMEM, EDTA, and FBS were purchased from Gibco. Aprotinin, bestatin hydrochloride, bromophenol blue, geneticin, glycine, leupeptin, NaCl, EGTA, penicillin, pepstatin A, PDL, protein A sepharose, SDS, streptomycin, sucrose, Tris-HCl, Triton X-100, HEPES, 3-[(3-Cholamidopropyl)dimethylammonio]-1-propanesulfonate, 4–2(aminoethyl) benzenesulfonyl fluoride hydrochloride (AEBSF), and L-α-phosphatidylcholine (PC) were obtained from Sigma-Aldrich. Geneticin was supplied by Invitrogen. The silver staining kit and MeOH were purchased from Wako Pure Chemicals. E-64 protease inhibitor was obtained from Calbiochem. Anti-P2X_4_ receptor antibody was supplied by Alomone Labs. Brain-derived phosphatidylserine (PS) was obtained from Avanti. Native mark (Invitrogen) microdialysis rods were purchased from Hampton Research. The spectrapor dialysis membrane was obtained from Spectrum Lab. n-octyl-β-D-glucopyranoside (βOG) was obtained from DOJINDO.

### Cell culture and establishment of stable P2X_4_R-expressing cell.

Human astrocytoma 1321N1 cells were maintained in DMEM, containing 5% (v/v) FBS, 100 μg/ml penicillin, and 100 μg/ml streptomycin (Sigma). For 1321N1 cells expressing P2X_4_R, 400 μg/ml G-418 (geneticin) was added. cDNA encoding rat P2X_4_R was subcloned into the pcDNA3.1 vector. Transfection was carried out with Superfect (QIAGEN) according to the manufacturer's protocol. 1321N1 cells successfully expressing P2X_4_R were confirmed by the ATP-induced increase in [Ca^2+^]i, and were isolated and proliferated.

### P2X_4_ receptor protein purification.

P2X_4_R-expressing 1321N1 cells were cultured to confluence and then harvested by scraping. The cells were homogenized with a Teflon homogenizer in HEPES buffer containing 20 mM HEPES, (pH 7.4), 320 mM sucrose, 5 mM EDTA, 5 mM EGTA, and protease inhibitors (100 μM AEBSF, 80 nM aprotinin, 5 μM bestatin, 1.5 μM E-64 protease inhibitor, 2 μM leupeptin, and 1 μM pepstatin). Supernatants obtained by centrifuging the homogenate at 3,000*g* for 15 min at 4 °C were further spun at 38,400*g* for 15 min to obtain membrane pellets. The pellets were resuspended in buffer containing 20 mM HEPES, (pH 7.4), 1% CHAPS, 100 mM NaCl, 5 mM EDTA, 5 mM EGTA, and protease inhibitors. The sample was treated with anti-P2X_4_R antibody (10 μg) and incubated for 24 h at 4 °C with gentle agitation. Then protein A sepharose (1 mg) was added to the sample and incubated for 1 h at 4 °C. The sample was then centrifuged at 3,000 *g* for 5 min and the pellets were washed with buffer (20 mM HEPES, [pH 7.4], 100 mM NaCl, 5 mM EDTA, 5 mM EGTA, and protease inhibitors) three times and treated with 50 μl 0.1 M glycine-HCl (pH 2.7) to dissociate the P2X_4_R protein from the antibody. The supernatant was transferred to a new tube and added to 1/10 volume of 1M Tris-HCl (pH 8.5).

### Native-PAGE and silver staining of purified P2X_4_ receptor protein.

Purified protein was resolved in a native sample buffer (62.5 mM Tris-HCl, [pH 6.8], 15% glycerol, 1% deoxycholate, and 0.01% bromophenol blue) and was loaded onto 4%–13% acrylamide gradient gel. Native Mark was used as a marker for detecting the molecular weight of purified P2X_4_R. After native-PAGE, silver staining was undertaken following the manufacturer's protocol (Silver stain kit II, Wako). After electrophoresis, the gel was transferred into a container and fixed with a first fixation buffer (10% MeOH, 10% acetic acid and 40% H_2_O) for 10 min followed by a 10-min second fixation in a second fixation buffer (10% fixation buffer A and 90% H_2_O). Then the gel was incubated in an intensification buffer (5% intensification buffer, 47.5% MeOH, and 47.5% H_2_O) for 10 min and washed with H_2_O for 5 min. The gel was stained in a stain buffer (5% stain solution A, 5% stain solution B, and 90% H_2_O) for 15 min. After washing with H_2_O (3 min × three times), the gel was incubated in a developing buffer (5% developing solution and 95% H_2_O) until the protein bands became visible.

### SDS-PAGE.

Cells and purified P2X_4_R protein were lysed with lysis buffer (containing 10 mM Tris, [pH 7.5], 150 mM NaCl, 1 mM EDTA, 1 mM EGTA, 1 % Triton X-100, 0.1% SDS, 1 mM sodium orthovanadate, 1% doxycholate, and 10 μg/ml each of aprotinin, bestatin, pepstatin A, leupeptin). For SDS-PAGE, the lysates were mixed with an equal amount of Laemli sample buffer (62.5 mM Tris/HCl, 20% glycerol, 2.5% SDS, 0.01% bromophenol blue, and 10% 2-merchapt EtOH) and then boiled at 95 °C for 5 min. Proteins were separated in 4%–13% acrylamide gradient gel and then visualized by silver staining. For western blotting analysis, electrophoresed proteins were transferred to the PVDF membrane and P2X_4_R protein was detected with anti-P2X_4_R antibody.

### Fast-scanning AFM.

The AFM experiments were performed using an NVB500 high-speed AFM (Olympus Corporation). BL-AC7EGS-A2 cantilevers with a spring constant of 0.1 N/m (Olympus Corporation) were used in the tapping mode with an oscillation frequency of 800–1,000 kHz. PDL (0.1 mg/ml in H_2_O) was treated on mica for 30 min at RT. The sample was washed with imaging buffer A (25 mM Tris-HCl, [pH 8.0], 137 mM NaCl, 2.7 mM KCl), and then deposited on the mica and incubated for 30 min at RT. For the time-lapse imaging of the P2X_4_Rs, mica rinsed with a high-salt buffer (10 mM Tris-HCl, [pH 8.0], 1M KCl, 30 min at RT) without PDL coating and imaging buffer B (25 mM Tris-HCl, [pH 7.4], 137 mM NaCl, 2.7 mM KCl) were used. To observe P2X_4_Rs in the presence of Ca^2+^, imaging buffer C (25 mM Tris-HCl, [pH 7.4], 137 mM NaCl, 2.7 mM KCl, 2 mM CaCl_2_) was used. To activate the P2X_4_Rs, caged ATP (200 μM) was uncaged by UV illumination (BH2-RFL-T3, Olympus). ATP and caged ATP were dissolved in imaging buffers A and B, respectively. Images containing 192 × 144 pixels were obtained at a scan rate of 0.2 or 0.5 fps for static images and 2.0 fps for time-lapse imaging.

### Processing of AFM images.

All AFM images were processed using Image J software (http://rsb.info.nih.gov/ij/). The P2X_4_R diameters were measured by using “segmented line selections.” The height and diameter were measured by using “Analyze-Plot profile” found on the menu bar. The 3-D images of P2X_4_R shown in Figure S1 were converted from 2-D AFM images with the Image J plug-in “interactive 3D surface plot” (http://rsbweb.nih.gov/ij/plugins/surface-plot-3d.html). The plug-in programs were downloaded from the Image J software homepage (http://rsb.info.nih.gov/ij/plugins/index.html). The P2X_4_R images were averaged with EMAN software [30] (http://blake.bcm.tmc.edu/eman/). Because the majority of the P2X_4_Rs exhibited a similar direction, we simply selected the P2X_4_R particles at random for averaging. All the P2X_4_R images used for EMAN processing were converted to TIFF files. The TIFF images were opened by boxer program and particles for averaging were selected. The selected images were processed with an averaging command in proc2d program. The resulting averaged image was saved in PNG file format. For 3-fold symmetrized averaging the P2X_4_Rs were rotated through three angles (0, 120, and 240°) with illustrator CS software and the file was converted to a TIFF file. The resulting three P2X_4_Rs were further averaged using EMAN software.

### Criteria for determining size parameters and selection of P2X_4_R particles for analysis.

We first established criteria for determining the P2X_4_R particle center. Three types of P2X_4_Rs were observed in the control, namely those with triangular, circular, and ellipsoidal structures (Figure S1A). The center of the triangular P2X_4_R was defined as the center of a triangular circumcircle. The center of the circular P2X_4_R was defined as the center of an approximated circle. The center of the ellipsoidal P2X_4_R was defined as the intersection of the long and minor axes. The center of the trimeric P2X_4_R was defined as the center of a circle connecting the highest points of all subunits. Next, we established P2X_4_R size criteria. In the present study, we defined the P2X_4_R dimensions as diameter and height. The diameter of the triangular and circular P2X_4_Rs was defined as the diameter of the circles used for determining the particle center. The diameter of the ellipsoidal P2X_4_R was defined as the average value of the long and short axes. The diameter of the trimeric P2X_4_R was defined as the diameter of a circle that circumscribed the three lobes. The particle height in the control was simply defined as the distance between the top of the particle and the mica surface. The height of the trimeric P2X_4_R was defined as the average height of three lobes. The height of P2X_4_R in a lipid bilayer was defined as the total distance from the top of the particle to the membrane surface plus the height of the lipid bilayer (4 nm). Then, we established criteria for particle selection. The P2X_4_R diameters obtained from 600 particles including activated and nonactivated P2X_4_Rs exhibited a clear single distribution and the top 5% and bottom 5% of the particles were eliminated from the analysis. The remaining 90% of the particles indicated by the arrows in Figure S1C(i) were used for analysis. In addition to this, P2X_4_Rs that exhibited a large noise (Figure S1C[ii]) were also eliminated from the analysis. During AFM observation, the P2X_4_Rs did not always provide clear images. Although the P2X_4_Rs had a clear topology in some frames, it was not clear in others. When the ATP stimulated P2X_4_Rs were selected for averaging, P2X_4_R particles without subunit-like structures were eliminated from the averaging process.

### Procedure for averaging P2X_4_Rs.

We performed the averaging in accordance with an early study [18]. First, we selected individual P2X_4_R particles on the basis of our criteria and then averaged them using EMAN software (nonsymmetrized averaging). Under our conditions, most of the P2X_4_Rs exhibited similar directions, so we did not perform any additional processing before averaging. The resulting images were further rotated (0, 120, and 240°) and averaged again (3-fold symmetrized averaging). When the activated P2X_4_Rs were averaged, the P2X_4_Rs without the subunit-like structures observed in the control were eliminated.

### Reconstitution of purified P2X_4_ receptors into artificial lipid membrane.

Lipid mixtures (100 μl) for reconstitution were prepared from L-α-phosphatidylcholine/brain-derived phosphatidylserine (PC/PS = 1:1, 160 μM) with 160 mM n-octyl-β-D-glucopyranoside. Mixed micelles were added to 100 μl of 100 ng/ml P2X_4_R protein. Detergent was removed by dialysis using microdialysis rods and a Spectrapor dialysis membrane (molecular cut-off of 50,000) in a dialysis buffer (30 mM HEPES, 5 mM EDTA, 1 mM EGTA, 0.02% of NaN_3_). The P2X_4_Rs were dialyzed for 5 d and the buffer was changed every day.

### DNA purification from primary cultured rat cortex astrocytes.

Purified DNA was prepared from primary cultured rat cortex astrocytes using ISOGEN (Nippongene). Primary rat cortex astrocytes were cultured as described in detail in our previous work [31]. DNA isolation was performed in accordance with the manufacturer's protocol. Confluent cultured astrocytes in a 100-mm cell culture dish were washed three times with PBS and lysed with 1 ml of ISOGEN. After homogenization by pipetting, the cell lysate was transferred to a 1.5-ml tube. Then 0.2 ml of chloroform was added to the tube and the resulting mixture was incubated for 3 min at RT after vigorous shaking (15 s). The tube was centrifuged (12,000*g*) for 15 min at 4 °C and the inter/organic phases were transferred to a new tube. Next, ethanol (0.3 ml) was added to the tube and incubated for 3 min at RT. The tube was centrifuged (2,000*g*) for 5 min at 4 °C. The supernatant was discarded and 1.0 ml of 0.1 M sodium citrate (in 10% ethanol) was added to the tube. After 30 min incubation at RT, the tube was centrifuged (2,000*g*) for 5 min at 4 °C. The precipitate was mixed in 2 ml of 75% ethanol and incubated for 30 min at RT. The tube was then centrifuged (2,000*g*) for 5 min at 4 °C. The precipitate was dried and dissolved in H_2_O.

### Calcium and dye uptake imaging of membrane reconstituted P2X_4_ receptors.

Calcium and dye uptake imaging of P2X_4_Rs was performed using a 500-μm hole cut in a plastic plate consisting of the bottom plate of a 60-mm cell culture dish. A Terumo syringe (25 gauge, 500 μm in diameter) was briefly heated with a gas burner and then pushed through the plastic plate. The resulting plastic burr around the hole was removed with a razor. Then 0.2 μl of imaging buffer containing 50 μM fluo-3 and 100 ng/μl DNA was placed in the hole. The top and bottom of the hole were covered by 1 μl of n-decane containing 2 mM PC/PS (1:1) and incubated for 5 min at RT. P2X_4_R-containing proteoliposome (0.5 μl) was supplied to the top surface of the hole and incubated for 10 min at RT. The bottom surface of the hole was covered with 2 μl of 10 mM Tris-HCl buffer (pH 7.4). Proteoliposome-containing buffer was carefully washed with 2 μl of 10 mM Tris-HCl buffer (pH 7.4) and then with 10 mM Tris-HCl buffer containing 20 μM EtBr with/without 2 mM CaCl_2_ (calcium imaging buffer or EtBr uptake imaging buffer). To stimulate the P2X_4_Rs, 1 μl of ATP (300 μM, the final concentration of ATP in the buffer was 100 μM) was added to the top of the hole. Calcium and dye uptake imaging was performed using a Zeiss LSM510 and ZEN2007 imaging system under a 5× objective. Throughout the functional analysis, fluo-3 was excited with the 488-nm line of an argon ion laser and the emitted light was collected using a 500–530-nm band-pass filter. EtBr was excited at 488 nm and the emission fluorescence was collected using 560–615-nm band-pass filters [32].

### Statistical analysis.

Average results are expressed as the mean ± SEM. Data were analyzed with the Student's *t-*test to determine the differences between groups. Significance was accepted when *p* < 0.05.

## Supporting Information

Figure S1 Criteria for Determining the Center, Diameter, and Height of P2X_4_Rs (A) Diagrams illustrating the criteria for the center of a P2X_4_R particle. (i) The center of a triangular P2X_4_R was defined as the center of a triangular circumcircle (left). The center of circular P2X_4_R was defined as the center of an approximated circle (middle). The center of an ellipsoidal P2X_4_R was defined as the intersection of the long and minor axes (right). The center of a trimeric P2X_4_R was defined as the center of a circle connecting the highest points of three lobes. (ii) Experimental examples of determining the particle center.
(B) Criteria for P2X_4_R size. The diameters of triangular and circular P2X_4_Rs were defined as the diameter of the circle used for determining the particle center. The diameter of an ellipsoidal P2X_4_R was defined as the average value of the long and short axes. (i) The diameter of a trimeric P2X_4_R was defined as the diameter of a circle that circumscribed the three lobes. (ii) Criteria for P2X_4_R height. In the control, the particle height was simply defined as the height from the mica surface (left). The height of a trimeric P2X_4_R was defined as the average value of the heights of the three lobes (right).
(C) Criteria for particle selection. (i) Size filtering. The P2X_4_R diameters obtained from 600 particles including activated and nonactivated P2X_4_Rs exhibited a clear single distribution and the top 5% and bottom 5% of the particles were eliminated from the analysis. The remaining 90% of the particles indicated by the arrows were used for the analysis. (ii) P2X_4_Rs that exhibit large noise were also eliminated from the analysis.
(D) Procedure for averaging of P2X_4_Rs. First, individual P2X_4_R particles were selected on the basis of our criteria and averaged using EMAN software (nonsymmetrized averaging). Under our conditions, most P2X_4_Rs exhibited similar directions, so we did not perform additional processing before averaging. The resulting images were further rotated (0, 120, and 240°) and averaged again (3-fold symmetrized averaging).
(2.02 MB EPS)

Figure S2 Nonsymmetrized Images of ATP-Induced Structural Changes in P2X_4_Rs Before activation, P2X_4_R was circular in shape and exhibited some fluctuation (−2.5 s to ≈0.0 s). After uncaging, P2X_4_R adopted a trimer structure within 0.5 s. Then, P2X_4_R exhibited a further structural change and adopted a pore dilation-like conformation. Ten P2X_4_R particles were averaged for each frame. Scale bar, 10 nm. AFM observation was performed in AFM imaging buffer B.
(957 KB EPS)

Figure S3 Another Example of Time-Lapse Imaging of ATP-Induced Structural Changes of P2X_4_R Before activation, P2X_4_R was circular (≈0.0 s). After uncaging, P2X_4_R changed its structure to a tripartite topology. Then, P2X_4_R exhibited a further structural change and adopted a pore dilation-like conformation. Ten P2X_4_R particles were averaged for each frame. Scale bar, 10 nm. AFM observation was performed in AFM imaging buffer B.
(693 KB EPS)

Figure S4 ATP-Induced Structural Changes in a Single P2X_4_R Several examples are shown of ATP-induced structural changes in single P2X_4_Rs (example 1–4) . After ATP binding, the individual subunit-like structures in single P2X_4_Rs become clearer. Scale bar, 10 nm.
(891 KB EPS)

Figure S5 AFM Images of Single P2X_4_Rs under 0-mM and 2-mM Ca^2+^ Conditions (A) (i) In the absence of Ca^2+^, clearly disengaged subunits in P2X_4_R were observed. (ii) Under 2-mM condition, P2X_4_Rs exhibited tripartite topology but were close together.
(B) Images of nonsymmetrized averaging at 0 mM (left) and 2 mM Ca^2+^ (right). Scale bar, 10 nm.
(784 KB EPS)

Video S1 Time-Lapse Imaging of ATP-Induced Structural Changes in P2X_4_R Before activation, the P2X_4_R was circular and exhibited some fluctuation (−2.5 s to ≈0.0 s). Caged ATP (200 μM) was uncaged at 0 s. After uncaging, the P2X_4_R adopted a trimer structure within 0.5 s. Then, the P2X_4_R exhibited a further structural change and adopted a pore dilation-like conformation. Ten P2X_4_R particles were averaged for each frame.
(989 KB AVI)

Video S2 Ca^2+^ Imaging of P2X_4_Rs under 2-mM Ca^2+^ Conditions The green fluorescence intensity derived from fluo3/Ca^2+^ after ATP (100 μM, at 0 s) increased immediately upon ATP addition.
(836 KB AVI)

Video S3 EtBr Uptake of P2X_4_Rs under 0 mM Ca^2+^ Conditions Under 0 mM Ca^2+^ conditions, the red fluorescent (DNA/EtBr) intensity gradually increased after ATP addition.
(817 KB AVI)

## Abbreviations

AFM: atomic force microscopy
ECD: extracellular domain
EM: electron microscopy
EtBr: ethidium bromide
FRET: fluorescence resonance energy transfer
ICD: intracellular domain
Panx1: Pannexin-1
PDL: poly-D-lysine
P2XR: P2X receptor
RT: room temperature
SEM: standard error of the mean
TMD: transmembrane domain
